# Physico-Chemical, Textural, Antioxidant and Sensory Characterization of White Chocolate Enriched with Barley Powder

**DOI:** 10.3390/foods15091548

**Published:** 2026-04-29

**Authors:** Otilia Cristina Murariu, Florin Daniel Lipsa, Irina Gabriela Cara, Gianluca Caruso

**Affiliations:** 1 Department of Food Technologies, ‘Ion Ionescu de la Brad’ Iasi University of Life Sciences, 700490 Iasi, Romania; 2 Research Institute for Agriculture and Environment, ‘Ion Ionescu de la Brad’ Iasi University of Life Sciences, 700490 Iasi, Romania; irina.cara@iuls.ro; 3 Department of Agricultural Sciences, University of Naples Federico II, 80055 Portici, Italy; gcaruso@unina.it

**Keywords:** *Hordeum vulgare* L., innovative functional food, fiber, color, polyphenols, carotenoids, chlorophylls, mineral composition

## Abstract

The enrichment of chocolate with healthy beneficial ingredients represents an effective strategy to create functional food with high nutritional and bioactive potential. Comparisons were made between eight treatments derived by the factorial combination of 2 types of butter (milk and cocoa) and 4 concentrations of green barley powder addition (1%, 3%; 5%; and 7%), plus 2 untreated controls (milk butter and cocoa butter with no green barley powder addition), in terms of chemical, colorimetric, physical, antioxidant, mineral and sensory characteristics of white chocolate. Increasing addition of green barley to both milk and cocoa butter led to the decrease in dry matter, soluble solids, pH and fat in the produced chocolate, with the untreated controls always showing the highest values. Opposite trends were recorded for proteins, fiber, ash and mineral substances. The ‘L’, ‘a’ and ‘b’ color components gradually decreased from the untreated control to the highest concentration of barley powder addition both to milk and cocoa butter. The increasing integration of barley powder either into milk or cocoa butter resulted in the gradual decrease in F max compression and F max cutting of the chocolate manufactured, compared to the untreated control. The addition of barley powder to milk and cocoa butter elicited a gradual increase in all the antioxidants analyzed, i.e., vitamin C, carotenes, lycopene and xanthophylls, and of chlorophyll a and b, compared to the untreated control. Vegetal flavor attributes were enhanced by the increasing addition of green barley powder. The latter incorporation into milk and cocoa butter sheds light on the interesting topic of conceiving and applying the manufacture of innovative functional chocolate with high content of fiber, nutrients and antioxidants.

## 1. Introduction

Increasing demand is being registered worldwide for healthy aliment with high nutritional and antioxidant value, named functional food [1]. The latter definition refers to the benefits provided by the mentioned aliment to one or more human body pivotal functions in adequate nutritional conditions, thus improving health status and/or reducing the risk of disease, but not ingested as a dietary supplement form [2].

*Hordeum vulgare* is a valuable source of soluble and insoluble dietary fiber and bioactive compounds, such as vitamin E, B-complex vitamins, enzymes, minerals, and polyphenols [3]. Barley powder is a functional ingredient with several health benefits also due to its high content of the polysaccharide β-glucan which is classified as a soluble dietary fiber [4], able to lower blood cholesterol [5] and glycemic index [6], controlling cardiovascular disease [7], lowering blood pressure, reducing fat absorption rate, and improving gastrointestinal activity in human beings [8]. In this respect, the integration of green barley powder into innovative production chains showed advantageous effects, like in dairy products [9], bread and beverages [10]. So far, there are not enough results regarding the integration of green barley powder into milk or cocoa butter to produce chocolate, but innovative research is needed within the project perspective of New Product Development (NPD), which is essential to the efficiency of the related organizing institutions. Chocolate derived from cocoa or milk butter is manufactured from cocoa butter, milk or milk products and sugars, containing at least 20% cocoa butter and 14% dry milk solids derived from partially or totally dehydrated whole milk, semi- or full-skimmed milk, cream, or from partially or completely dehydrated cream, butter or milk fat, at least with 3.5% of milk fat presence, according to the Directive 2000/36/EC [11]. However, a high number of antioxidants, either as direct compounds or plant extracts, have been added in previous works to create functional foods [12,13,14,15,16,17]. Notably, most research targeting innovative chocolate production strategies has been focusing on the antimicrobial and antioxidant activities of plant polyphenol extracts to prevent diseases [18], as well as on the nutritional and bioactive contribution of plant material such as barley powder to obtain health beneficial and economically sustainable aliments. New functional foods have modified the related industry proposing appealing perspectives of sensory, physical, and nutritional attributes to meet consumers’ demand [19].

Functional foods are termed “natural or processed foods containing effective and non-toxic amounts of bioactive compounds, which provide a clinically proven and documented health benefits through the use of specific biomarkers to prevent human aging, treat chronic diseases or their symptoms” [20].

The present research developed a strategy targeting to create sustainable systems to manufacture innovative functional food with enhanced nutritional and antioxidant properties, within the chocolate industry, allowing to fulfil either the projected world inhabitant augmentation or consumers’ willingness to avail healthy aliments produced by incorporating plant-derived beneficial ingredients such as barley, also fostering the profits of related chains [1].

This study was aimed at valorizing green barley powder to evaluate the most efficient technological practices and recipes to manufacture new chocolate rich in nutrients and phytochemicals. In this respect, it was expected that increasing the incorporation percentage of the mentioned plant material into milk and cocoa butter would have a variable impact depending on the chocolate characteristics examined, i.e., chemical, color, physical, antioxidant, mineral and sensory.

## 2. Materials and Methods

### 2.1. Experimental Protocol and Raw Materials

Research was carried out at ‘Ion Ionescu de la Brad’ Iasi University of Life Sciences (Romania) in 2025.

Comparisons were made between eight treatments derived by the factorial combination of 2 types of butter (milk and cocoa) and 4 concentrations of green barley powder addition (1%, 3%; 5%; and 7%), plus 2 untreated controls (milk butter and cocoa butter with no barley powder addition), in terms of chemical, colorimetric, physical, antioxidant, mineral and sensory characteristics of chocolate. A complete block randomized design was used with three replicates.

### 2.2. Barley Material and Chocolate Preparation

Green barley (*Hordeum vulgare* L.) powder with 100–150 µm particle size and 13.8% humidity, obtained from organically grown young barley leaves, manufactured by Niavis (Brasov, Romania), was used in this study as raw material to produce innovative chocolate products, following storage in dry and cool conditions.

Chocolate processing was performed including green barley powder in the recipes, as previously described [21].

Chocolate quality characteristics are the consequence of physical and biochemical processes occurring during manufacturing, starting from the main ingredients such as powdered milk mass, barley powder, cocoa butter, sugar and flavorings, aiming at obtaining a product with solid components smaller than 20–25 µm which is the threshold for olfactory organ detection [21].

The chocolate samples corresponding to the 8 experimental treatments applied, plus 2 untreated controls, were manufactured in the shape of bars, as can be observed in the below images.


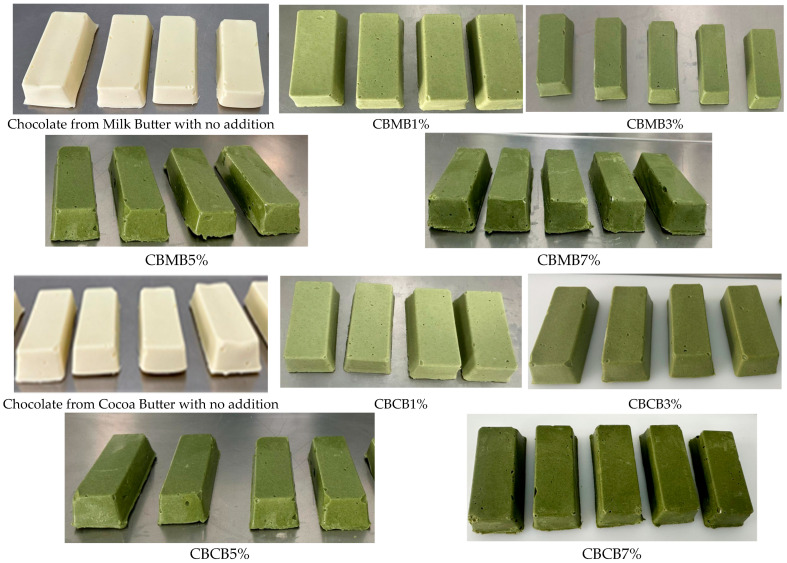


Referring to the international classification of chocolate [22], a 30% sugar was included in the recipe for manufacturing the barley integrated chocolate samples, in addition to 26% fat in powdered milk and 15% fat in cocoa butter (Table 1). In this respect, chocolate quality characteristics (unctuousness, solid particle dispersion in butter, taste, smell) relate to barley powder particle fineness, all the mentioned ingredients and flavorings.

### 2.3. Determination of Chemical, Color and Textural Parameters

Total dry matter, soluble solids, pH, fat, proteins, fiber, ash and mineral substances of chocolate samples were determined in compliance with AOAC procedures [23]: particularly, dry matter was assessed at 103–105 °C until constant weight in a forced air-drying oven (Biobase^®^, Jinan, China); fiber according to the Fist Action 2022.01.

Color was measured as reported in Milovanovic et al. [24]. The color components of chocolate samples were determined in replicate using a Konica Minolta CR-400 trichomatric reflectance colorimeter with Spectra Magic NX 1.3 software (Konica Minolta Sensing INC.^®^, Osaka, Japan), at an ambient temperature. The results were expressed according to the CIE Lab system components L*, a*, b*. Measurements were made with an 8 mm optical glass aperture, upon calibrating the equipment using a standard white plate. The samples were evenly leveled and placed in a clear glass container for colorimetric analysis. The measurements were repeated three times at distinct points on the sample to ensure the accuracy of the results.

Maximum compression and cutting strength were determined according to Wee et al. method [25], using a Mark 10^®^ texturometer (Mark-10 Corporation, Copiague, NY, USA) equipped with a TA5 type cylindric probe with 12.7 mm diameter and 35 mm height with a 100 range and a 0.05 N resolution, and a Warner Bratzler V-knife (Lloyd Instruments Ltd., Bognor Regis, UK) to cut the samples, with the insertion velocity into the sample of 200 mm per minute; the software MeasurePlus v15 and GraphPad Prism v10 were used to record and interpret the graphs, respectively.

### 2.4. Extraction of Bioactive Substances from Chocolate Samples, and Determination of Antioxidant Activity, Vitamin C, Total Polyphenol, Carotenoid, and Chlorophyll Contents

Vitamin C, total polyphenols and carotenoids (carotenes, lycopene and xanthophylls) contained in the chocolate samples derived from the addition of green barley powder to milk and cocoa butter were extracted and analyzed, along with the antioxidant activity, as previously described [21], as well as chlorophylls a and b [26]. Particularly, the phytochemicals were extracted using the ultrasound-assisted method: one g of sample was mixed with 70% ethanol or 10 mL of n-hexane/acetone solvent mixture (carotenoids) and subjected to an ultrasound treatment for 30 min at a maximum of 32 °C and 40 kHz frequency. The resulting crude extract was centrifuged for 10 min at 5000 rpm at 4 °C; the supernatant was collected after separation, and the planned determinations were made.

### 2.5. Determination of Mineral Elements

The content of mineral elements (K, Ca, Mg, Na, P, Zn, Cu, Fe) was measured, according to the literature reports [21], by the atomic absorption spectrometry using the ContrAA700 Analytik Jena (Jena, Germany) with a flame atomizer system, expressing the results in mg 100 g^−1^ d.w., with 3 biological and 2 technical repetitions.

One g of each chocolate sample was mineralized at 190 °C for 30 min by a mixture of HNO_3_ and H_2_O_2_ (7:3), using a MiniWAVE Microwave (SCP Science, Baie-d’Urfé, QC, Canada) digestion system at 1000 W, equipped with a 50 mL Teflon vessel. After cooling, the samples were transferred into a 50 mL volumetric flask and diluted with ultrapure water until the mark. A blank sample was added in every digestion run, and each sample was prepared in triplicate.

### 2.6. Evaluation of Sensory Features

The sensory evaluation test was performed on the chocolate samples associated with the 10 experimental treatments (including the 2 untreated controls) examined, by anonymous coding, as previously reported [21], applying a questionnaire to a 20-person (10 women and 10 men) panel, with spatial separation among individuals to prevent opinion exchange. This investigation was reviewed and approved by the Ethics Committee for Research in Human Beings of the Department of Food Technologies, Faculty of Agriculture, of the mentioned University, in compliance with the European Union Guidelines of Ethics and Food-Related Research [27].

The sensory analysis was performed using a structured questionnaire and a 10-point discrete scale (1 = lowest intensity/least pleasant; 10 = highest intensity/most pleasant). Panelists were instructed to assign only integer values. The evaluated attributes included visual characteristics (external and cross-sectional appearance, external and internal color, product thickness), textural and mouthfeel properties (particle fineness, consistency, mouthfeel, astringency), and overall acceptability. Additionally, texture perception during oral processing (mastication perception, breaking resistance, stickiness, and hardness) was assessed using structured intensity scales. A descriptive sensory component was also included, allowing the evaluation of the intensity of specific aroma (cocoa butter, animal fat, milk powder, vegetal/green notes) and taste descriptors (grassy, bitter, sweetness intensity, malt, green walnut), as well as the identification of undesirable sensations (rancid, acidic, bitter, tasteless).

Samples were coded with random three-digit numbers and presented in a random-sized order on white plates under controlled conditions (20–22 °C).

The mentioned multidimensional approach is consistent with current practices in chocolate sensory research, where both visual and textural attributes significantly influence consumer acceptance.

Particularly, the fineness of particles from the barley powder addition was explicitly assessed using a structured 10-point scale, because this parameter is critical in chocolate systems, as particle size distribution directly affects mouthfeel, smoothness, and perception of grittiness. By including this attribute, the study ensures that the incorporation of barley powder does not negatively impact the characteristic smooth texture expected in chocolate products.

Furthermore, the questionnaire integrates a detailed flavor and taste profiling section, allowing the identification and intensity grading of specific sensory notes such as cocoa butter, dairy, vegetal/green notes, malt, bitterness, and sweetness. This is particularly relevant for barley-enriched formulations, where vegetal and malty notes may emerge and influence product acceptability. In addition, the identification of undesirable sensations (e.g., rancid, acidic, excessively bitter, or flat taste) is essential for validating product quality and ensuring that the addition of barley powder does not introduce sensory defects.

A key strength of the methodology is the inclusion of dynamic texture perception parameters (mastication perception, breaking resistance, adhesiveness, and hardness), providing a deeper understanding of structural modifications induced by the barley powder and their impact on oral processing behavior.

Finally, the use of a 10-point hedonic scale across all attributes, combined with the evaluation of overall impression, enables robust statistical interpretation and comparison between samples with different levels of barley powder incorporation.

The scale used was discrete and structured, not continuous, and the panelists selected a single integer corresponding to their perception, with no intermediate scoring between integers allowed, ensuring consistency and facilitating statistical analysis.

To minimize potential bias, the samples were presented using a randomized serving order, which differed between panelists, to reduce order effects and sensory fatigue, in accordance with the standard sensory evaluation practices.

### 2.7. Statistical Analysis

Data were processed by analysis of variance (ANOVA) and mean separations were performed through Duncan’s test, with reference to a 0.05 probability level, using SPSS software version 30.

## 3. Results

### 3.1. Chemical, Color and Physical Parameters

Increasing addition of green barley to both milk and cocoa butter led to the decrease in dry matter, soluble solids, pH and fat in the produced chocolate, with the untreated controls always showing the highest values (Table 2); opposite trends were recorded for proteins, fiber, ash and mineral substances. No significant differences arose between milk and cocoa butter on average.

In previous research [9], the addition of 15% barley flour to Rayeb milk increased the levels of dry matter, carbohydrates, proteins, ash and antioxidant activity.

Barley is a raw material rich in dietary fiber, protein, and minerals, with a lower lipid content compared to chocolate, whose fat fraction is therefore reduced upon the integration of barley powder which acts as a fortifying ingredient increasing proteins and minerals; the fiber components of *H. vulgare* generally include significant amount of β-glucans with beneficial physiological effects on carbohydrate and lipid metabolism, enhancing the nutritional value [28] as well as the functional and physical properties of food [29].

The decrease in dry matter and soluble solids suggests that the barley powder introduced into the matrix has a higher content of hydrophilic substances, fiber and non-starch polysaccharides, possibly increasing water retention, and a lower level of soluble substances.

The decrease in fat content and augmentation in structural components with functional potential have a favourable impact on chocolate, though from a technological and legislative perspective the reduction in lipids may become a limiting factor for the product’s commercial classification if labeled as “milk chocolate”, according to the Directive 2000/36/EC [30]. The latter indicates a minimum total fat content of 25% for “milk chocolate”, along with minimum thresholds for total solids and cocoa butter, which are met by the control samples and the experimental treatments with the lowest additions in the present research.

Jevcsák et al. [31] showed that the addition of *Agaricus bisporus* powder significantly increased the protein, dietary fiber, and mineral content of chocolate, while maintaining a satisfying sensory acceptability. Similarly, Monteiro et al. [32] reported that adding baobab pulp to dark chocolate increased the content of some minerals and improved the product’s functional profile, consistent with current research on functional chocolate [33].

In our research, no significant differences were generally recorded between cocoa and milk butter referring to fats, proteins, fiber, and minerals, suggesting similar integration of barley powder into the two matrices.

The addition of plant extracts of *Sambucus nigra* and *Aronia melanocarpa* into chocolate enhanced antioxidant activity, moisture, fat content and viscosity [34,35].

The incorporation of seaweed extracts of spirulina and kelp into chocolate created a functional food rich in carbohydrates, fatty acids, amino acids, nutrients, vitamins and antioxidants [36,37].

The ‘L’, ‘a’ and ‘b’ color components gradually decreased from the untreated control to the highest concentration of barley powder addition both to milk and cocoa butter (Table 3). The ‘L’ and ‘a’ color components were higher in the untreated control derived from cocoa butter than in that associated with milk butter, whereas the highest barley powder addition to milk butter led to a higher ‘b’ color component compared to the corresponding concentration integrated into cocoa butter.

The increasing incorporation of barley powder either into milk or cocoa butter resulted in the gradual decrease in F max compression and F max cutting of the white chocolate manufactured, compared to the untreated control (Table 3). Moreover, the top barley powder addition led to higher F max compression, in comparison with the corresponding concentration integrated into cocoa butter.

The mentioned results show that the gradual addition of green barley powder to chocolate formulated with either milk butter or cocoa butter had significant effects on both color and mechanical strength, thus acting not only as a nutritional fortifier but also as a physical restructuring factor of the product lipid matrix.

The pronounced reduction of the ‘L’ component shows that the barley powder significantly diminished the creamy-white appearance specific to white chocolate and led to a progressive darkening of the surface. At the same time, the decrease in b* coordinate indicates a partial loss in the golden-yellow hue characteristic of the matrix rich in milk and cocoa butter, and the reduction in a* value suggests an augmentation of green intensity.

The content of pigments contained in the green barley powder added to the two butter types to produce chocolate influenced the trends of the ‘a’ and ‘b’ color components [38]; indeed, white chocolate is highly sensitive to plant-based additives because of the absence of cocoa solids not optically masking the new ingredients, which immediately becomes dominant in visual perception, as previously reported [39,40,41].

Our results are consistent with Okstaviyani et al. [39] findings about the concentration-dependent effect of plant-based powder incorporation into chocolate leading to a decrease in brightness; the color changes directly relate to the natural pigments of the added functional ingredient and their interaction with the fat matrix and dispersed chocolate phase.

Consumers are strongly attracted by the color of food products which depends on several factors among which are cultivar and ripening stage [42]; particularly, chocolate appearance includes some parameters, such as brightness, shape, surface smoothness, translucency and color [43,44]. The color components of food products are influenced by the type of material added [45,46] but Toker et al. [45] recorded narrow ranges of variation, similarly to previous findings [47]. A darker [45] or redder chocolate [48], compared to control, was elicited by EPA/DHA source integration, though the latter origin and form have an important impact [45], as well as the chocolate composition and the manufacturing conditions [49,50]. However, fat bloom development can worsen both the visual and textural quality of chocolate, thus reducing the effect of the EPA/DHA on the Whiteness Index [45].

Notably, from a quality standard perspective, EU legislation and Codex [30] do not set numerical thresholds for L*, a*, or b*, but define that a product can be marketed as white chocolate if contains at least 20% cocoa butter, 14% total milk solids and 3.5% milk fat or 2.5–3.5% according to Codex CXS 87-1981.

The decrease in F max compression and cutting under increasing barley powder addition reflects a reduction in structural firmness, suggesting that the crystalline fat matrix became less compact or less effective at retaining the solid phase with augmenting plant particle proportion. In this respect, Jevcsák et al. [31] showed that fortifying chocolate with *Agaricus bisporus* powder increasingly reduced firmness, compared to the control samples, upon rising fungus addition which caused continuous microstructure disruption in the chocolate. In other studies, Drosou and Krokida [51] recorded the simultaneous alteration of fat phase stability, crystal structure, and product mechanical response in white chocolate enriched with microencapsulated β-carotene.

In a previous study [52], the breaking strength of chocolate decreased with rising integration up to 10% of barley powder which increased the occurrence of solid phase particles. From a mechanistic point of view, the reduction in maximum compressive force in the barley-added chocolate can be elicited by plant particles disrupting the continuity of the crystallized fat network and reducing the degree of internal cohesion. In the same direction, vegetal extract application reportedly introduces hydrophilic compounds and fibers that alter moisture distribution and the fat/solid phase ratio [53] or, with the opposite trend, the product firmness is increased [54].

Toker et al. [45] investigated the enrichment of chocolate with encapsulated or microalgae containing forms of Eicosa-Pentaenoic-Acid (EPA) and Docosa-Hexaenoic-Acid (DHA), after the conching process. A shear-thinning behavior was recorded in chocolate samples, due to the structural breakdown consequent to the applied shear force [44] and the product constituent molecule alignment [55]. The integration of different EPA/DHA forms (microalgae/oil and powder) neither remarkably impacted chocolate plastic viscosity nor modified the manufacturing process [45].

Production chain efficiency is affected by chocolate physical properties [56] particularly referring to plastic viscosity [44], product surface, particle concentration and interactions, and emulsifiers which influence yield stress [46]; hardness depends on numerous conditions, i.e., ingredients, manufacturing, fat stability, with sugar-free chocolate commonly showing a softer structure than the traditional one [57]. Triple chain crystallization of fat triglycerides in cocoa butter form V is associated with much greater thermodynamic stability, compared to the double-chain [46], confirming the chocolate hardness–fatty acid profile connection [57]. Toker et al. [45] reported the influence of fatty acid composition on chocolate hardness, which was reduced by EPA/DHA addition, and the decrease in melting temperature with rising polyunsaturated fatty acid (PUFAs) content.

Tolve et al. [58] recorded a viscosity increase in No Palm No Sugar chocolate upon Mg-CaCO_3_ nanoparticle addition, with the Reference Brand (RB) product, added with palm oil and sugar, showing a higher viscosity than No Palm samples manufactured with sunflower oil and shea butter. Yield stress, highest in RB chocolate, plastic viscosity and consistency index displayed the same trends. The rheological behavior of molten chocolate is influenced by several connected factors, such as humidity, fat, emulsifier, particle size and shape, processing technique, and the fat content, which may lower yield stress and viscosity of the basic formulation [59]. Vitamin D incorporation into the product reduced interparticle interactions, thus influencing yield stress [60], presumably due to the higher fat-like material available to coat particles [58]; sample humidity consequent to nanoparticle use is also likely to encourage chocolate viscosity increase.

Viscosity was lowered in No Palm No sugar chocolate integrated with Vitamin D + calcium, compared to the only Ca addition [58], suggesting more interactions between particles not appropriately coated because of less fat available [61].

### 3.2. Antioxidant Properties

The addition of barley powder to milk and cocoa butter elicited a gradual increase in antioxidant activity and all the antioxidants analyzed, i.e., vitamin C, polyphenols, carotenes, lycopene, xanthophylls, chlorophyll a and b, compared to the untreated control (Table 4 and Table 5). The values related to the antioxidant activity and polyphenols were higher for all the experimental treatments associated with cocoa butter, compared to the corresponding ones of milk butter (Table 4); a similar trend was recorded for vitamin C from 3 to 7% barley powder addition, whereas the control and the lowest concentration did not significantly affect this antioxidant compound. As for carotenoids, the values of carotenes and lycopene were higher under the treatments from 3 to 7% barley powder concentration associated with milk butter, compared to those relevant to cocoa butter, whereas xanthophylls showed an opposite trend (Table 5).

The integration of green barley powder transformed white chocolate, a matrix low in bioactive compounds, into a product with a significantly improved antioxidant profile. The latter is beneficial because white chocolate contains few cocoa solids and, consequently, much lower antioxidants, compared to dark chocolate, which has arisen a growing interest in functional chocolates combining sensory acceptability with a higher density of bioactive compounds [62].

The transfer of bioactive compounds from the barley powder into the chocolate matrix is fully consistent with previous findings regarding barley sprouts/young barley richness in polyphenols, chlorophylls, and other compounds, like β-glucan, which is an important component of dietary fiber [63] with antioxidant activity, making them promising functional ingredients [64,65,66]. In our research, chocolate derived from cocoa butter with no barley addition showed higher antioxidant activity, vitamin C, polyphenol and xanthophyll levels than the untreated milk butter-based chocolate and, consequently, the same differences were recorded upon the integration of all barley concentrations; opposite trends were shown by carotenes and lycopene. The mentioned substances contribute to improving the antioxidant capacity of functional foods with beneficial effects on human health, such as anti-inflammatory and cardioprotective activity [67,68,69]. Previous studies on fortified products showed the important contribution of plant-based ingredient addition to enhance the polyphenol content and, accordingly, the antioxidant capacity in foods [70,71,72].

From a legislative perspective, according to the European framework on nutrition and health claims, functional claims may only be used if supported by remarkable levels of bioactive compounds and scientific evidence [33], and compounds with antioxidant activity must be characterized and quantified [73]. In this respect, enriching chocolate with green barley powder contributes to increasing its nutritional density and bioactive compound content, which may represent a promising strategy for developing functional confectionery products tailored to current consumer demands for health-promoting foods.

Referring to the described outcome, the higher percentage of barley powder addition resulted in a higher impact on chocolate polyphenol and antioxidant activity, due to the remarkable polyphenol content of the added material, as also found with other integration types [38].

In a previous investigation [74], the addition of yellow tea extract to dark, milk and white chocolates fostered the phenolic content and antioxidant activity.

Bolenz and Glöde [75] found a positive correlation between grape pomace added to chocolate, total phenol content and antioxidative capacity, with 3.5% by-product leading to 236 mg GAE 100 g^−1^ d.w., though chocolate naturally contains polyphenols deriving from cocoa particles. Phenolic content rose in foods incorporated with cinnamon, grape seeds and pomace in previous research [76,77]. A lower polyphenol increase along with a more neutral taste was elicited by grape seed addition, compared to the peels, leading to darker chocolate with an interesting fruity taste. Purified extracts proved to be an effective alternative to pomace products in previous investigations [76,78].

In previous research [58], the highest polyphenol concentration was recorded in Reference Brand chocolate enriched with vitamin D and magnesium–calcium carbonate nanoparticles, and the lowest level upon the integration of Vitamin D + Ca due to the low cocoa and high hazelnut content of chocolate spread [79]. The improvement of endothelial function and oxidative stress in patients with diabetes, because of high antioxidant content in extra virgin olive oil integrated chocolate, was recorded by other authors [80]. The polyphenol concentration was high in the Simulated Salivary Phase, decreased in the Simulated Gastric Phase and rose again in the Simulated Intestinal Phase [58], referring to the bioaccessibility of soluble compounds [81], similarly to the pH trend, as also reported in vegetable juices [82] and fruits [83]. Polyphenols are hydrolyzed by digestive enzymes and then absorbed, and their content and form in aliments can be affected by mechanical destruction, food matrix, processing techniques, gastrointestinal conditions, enzyme systems and interactions with other diet components [84]. Tagliazucchi et al. [85] recorded the grape polyphenol release in the Simulated Phases, i.e., about 50% in Salivary and 20% in Gastric and Intestinal ones. Hasni et al. [86] found the formation of protein–polyphenol complexes following milk casein and polyphenol interactions, in black tea extracts, possibly leading to a decrease in polyphenol bioaccessibility. In this respect, acid conditions may elicit the remarkable increase in matrix degradation during the Simulated Gastric Phase, whereas lipase activity may be the cause of the intestinal additional rise.

Wootton-Beard et al. [82] reported the enhancement of nutritional and functional properties of white chocolate upon the integration of *Moringa oleifera* leaf extracts.

The addition of different fruit extracts to chocolate improved health benefits, mainly referring to bioactive and nutrient substances [83,85]. Moreover, phenolic content and antioxidant activities were enhanced by incorporating into chocolate different ingredients, such as green tea extract [86], peanut peels [87], small fruit and pomegranate juice [48,88,89], extracts of elderberry and chokeberry plants [34,35] as well as seaweeds spirulina and kelp [36,37].

The positive effects of chocolate were recorded in decreasing cadmium-induced toxicity in mice, consequently diminishing DNA damage, apoptosis, cell necrosis, oxidative stress, and re-establishing mitochondria efficiency [90], in stabilizing the levels of memory, attention, and flexibility [91], and enhancing butyrate-producing bacteria growth, improving gut health [92].

### 3.3. Mineral Elements

The content of both the macro- and microelements measured in chocolate increased with rising the green barley powder addition either to milk and cocoa butter (Table 6); only potassium and calcium did not significantly differ between the experimental treatments applied. The highest levels of P, Cu and Fe were recorded in the chocolate derived from the 7% addition of barley powder to cocoa butter, whereas the same concentration integrated into milk powder led to the highest Zn accumulation in chocolate.

The mineral elements analyzed showed different trends because of adsorption and cation exchange laws related to the charge of the various cations: the bivalent ones have either a smaller ray or lower hydration degree, with consequent higher electrostatic field and therefore more intensely retained, compared to the monovalent.

K and Ca were not significantly influenced by the experimental treatments, suggesting that the barley powder contribution of these elements was partially offset by the baseline composition of the chocolate matrix. The latter situation is particularly referred to Ca which is appreciably present in milk butter, whereas Fe, Zn, Cu, and Mg were more responsive to the plant ingredient addition to chocolate, consistently with recent reports on food fortification by plant- or fungus-based ingredients [31,93]. Our results confirm that barley powder functioned as a mineral fortification ingredient, not merely as a source of antioxidant compounds [65,94].

Differences in element contents were recorded between the two lipid matrices examined referring to P and Cu which were higher under the highest barley integrations into cocoa butter, compared to milk butter, whereas the trend of Zn was opposite and that of Fe was controversial. The latter descriptions suggest a similar influence of the two fat phase types and the overall matrix compositions on the distribution and retention of minerals in the final product. Indeed, the added ingredients to chocolate interact with the fat phase, proteins, and sugar particles, which affect retention and analytical expression of minerals, i.e., the fortification degree, as previously highlighted [95].

From a functional standpoint, these elements are involved in essential physiological processes, such as the contribution of magnesium to energy metabolism and muscle activity, of iron to oxygen transport and fatigue reduction, of zinc to immune function and cell protection against oxidative stress, under the conditions and formulations authorized by EU legislation for health claims [33]. Therefore, enhancing the mineral profile by adding barley powder raises chocolate from the traditional hedonistic product rank to a nutritionally valuable functional food [95].

Recent studies revealed that young barley powder provides a significant contribution to the intake of macro- and microelements, including Fe and Mg, though the mineral composition also depends on cultivar as well as pre- and post-harvest management [65].

Minerals are essential nutrients with important functions related to metabolic activities and maintenance in humans. In a previous study [58], the addition of Mg Ca carbonate nanoparticles to chocolate increased calcium and magnesium content by nine and two times, respectively, compared to other treatments. The differences recorded depend on different factors, such as crop management [96] and formulation ingredients, as reported for the effect of hazelnut content in chocolate on K, P, Ca, Mg, B, Cu and Mn [97].

From the legislative point of view, in order to use nutritional claims like “source of minerals” within the EU Countries, the product must contain at least 15% of the Nutrient Reference Value (NRV) per 100 g for solid foods; this principle is derived from the framework related to nutritional claims and the NRVs set forth in EU legislation. The relevant NRVs are 2000 mg of K, 800 mg Ca, 375 mg Mg, 700 mg P, 14 mg Fe, 10 mg Zn, and 1 mg Cu. Therefore, the thresholds of 15% NRV 100 g^−1^ are approximately: 300 mg K, 120 mg Ca, 56.3 mg Mg, 105 mg P, 2.1 mg Fe, 1.5 mg Zn, and 0.15 mg Cu. All the chocolate samples analyzed in the present research met the mentioned criterion for magnesium and calcium, while the highest fortification allowed to fulfil the criterion also for iron and zinc; for copper, only the highest barley powder addition to cocoa butter reached the minimum threshold [30,33]. However, in terms of labeling, the EFSA guidance on antioxidant and health claims emphasizes that any constituent cited must be sufficiently characterized and quantified [73], and texture, sensory acceptability and storage stability should finalize the evaluation [98].

### 3.4. Sensory Features

**Figure 1:**
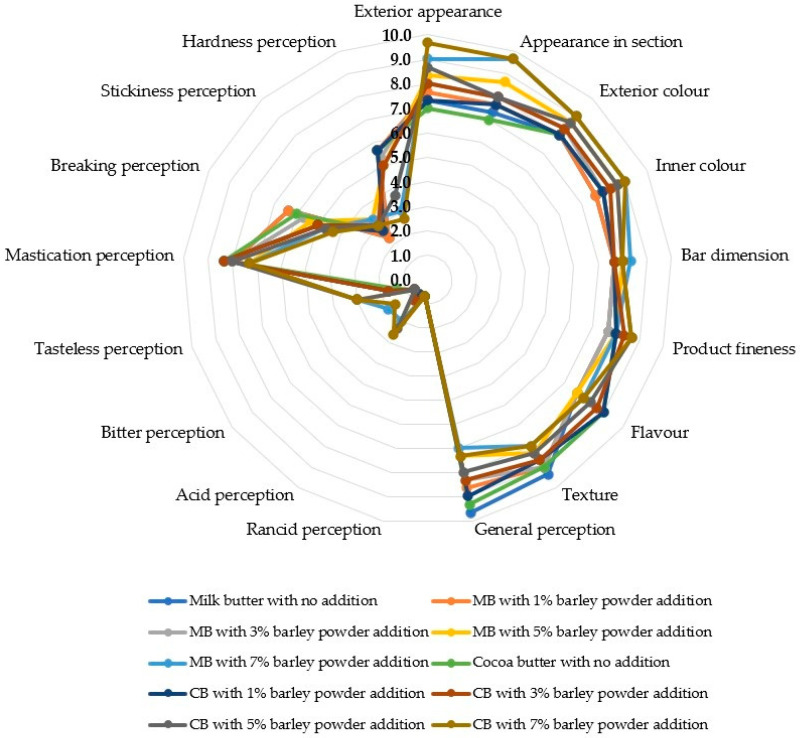
Sensory features of chocolate derived from the addition of green barley powder to milk and cocoa butter. CBMB1: chocolate from 1% barley powder addition to milk butter; CBMB3: chocolate from 3% barley powder addition to milk butter; CBMB5: chocolate from 5% barley powder addition to milk butter; CBMB7: chocolate from 7% barley powder addition to milk butter; CBCB1: chocolate from 1% barley powder addition to cocoa butter; CBCB3: chocolate from 3% barley powder addition to cocoa butter; CBCB5: chocolate from 5% barley powder addition to cocoa butter; cbcb7: chocolate from 7% barley powder addition to cocoa butter.

**Figure 2:**
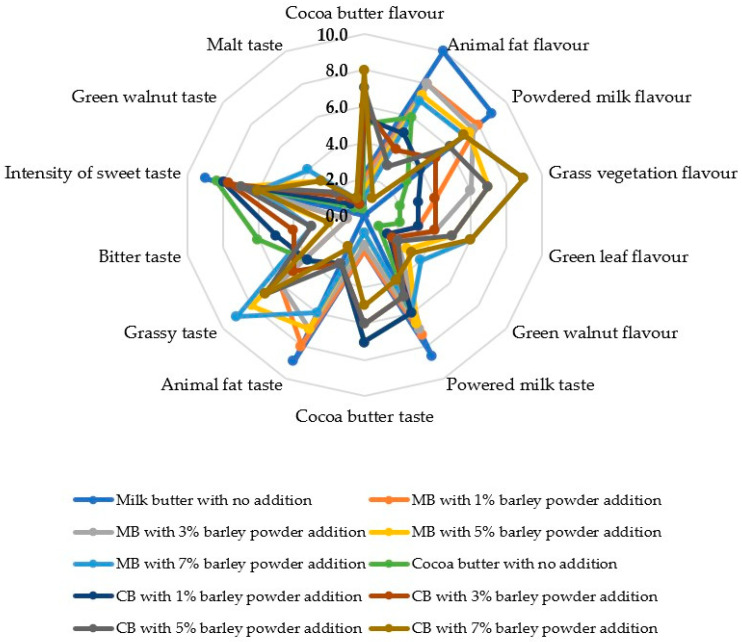
Sensory features of chocolate derived from the addition of green barley powder to milk and cocoa butter. CBMB1: chocolate from 1% barley powder addition to milk butter; CBMB3: chocolate from 3% barley powder addition to milk butter; CBMB5: chocolate from 5% barley powder addition to milk butter; CBMB7: chocolate from 7% barley powder addition to milk butter; CBCB1: chocolate from 1% barley powder addition to cocoa butter; CBCB3: chocolate from 3% barley powder addition to cocoa butter; CBCB5: chocolate from 5% barley powder addition to cocoa butter; CBCB7: chocolate from 7% barley powder addition to cocoa butter.

Most of the sensory features examined in the chocolate samples showed an increasing trend from the untreated control to the highest green barley powder addition of 7%, both in milk and cocoa butter derived products (Figure 1 and Figure 2; Table 7): exterior appearance, appearance in section, exterior and inner color, grass vegetation, green leaf and green walnut flavor, grassy, green walnut and malt taste, acid, bitter, tasteless and stickiness perception.

Other sensory features attained the highest levels in the untreated chocolates and decreased with rising the addition percentage of green barley powder either to milk or cocoa butter: Texture, general perception, animal fat flavor, powdered milk, cocoa butter, animal fat and intensity of sweet taste, mastication, breaking and hardness perception.

Finally, four sensory features showed controversial trends: flavor and bitter taste decreased with rising addition of green barley powder to milk butter but augmented with increasing integration of the mentioned material to cocoa butter; cocoa butter and powdered milk flavor displayed opposite trend (Figure 1 and Figure 2; Table 7).

In the present research, fortification with moderate addition of green barley powder did not strongly affect the visual characteristics of the product, which are essential for its acceptability, with the color and appearance being the first features evaluated by consumers [98]. Moreover, the mentioned integration level did not compromise flavor, texture and product fineness of chocolate, whereas the 7% addition slightly decreased the scores of texture, taste and overall perception due to a stronger influence of the plant particles on the chocolate matrix, consistently with previous findings [99,100].

The scores associated with negative taste attributes (perceived acidity, bitterness, or rancidity) were relatively low for all the experimental treatments examined, indicating that the green barley powder did not show evident effects on the mentioned characteristics. In this respect, maintaining sensory balance is a major challenge in the development of functional products obtained by plant-based ingredient integration [98].

Considering that the score related to general perception (overall acceptability) was high for most formulations, particularly those added with 1 to 5% barley powder, it can be inferred that the product resulted sensorially appealing after fortification. In this respect, fortified products are accepted by consumers if they are nutritionally beneficial even with moderate sensory changes [95].

Recent studies showed that chocolate is an excellent carrier for functional ingredients, as its lipid matrix can partially mask the taste of some bioactive compounds and facilitate the integration of plant-based ingredients or other nutrient sources [95,98].

In an investigation on chocolate moderately fortified with cactus powder, the overall acceptability scores were similar to the control ones, while the product’s antioxidant content significantly increased [100].

In the present research, the addition of barley powder primarily altered the aroma and flavor profile, rather than the basic attributes associated with sweetness or the classic notes of milk and cocoa butter. As the fortification level increased, a gradual decrease was observed in the descriptors typical of conventional white chocolate, such as cocoa butter flavor, powdered milk flavor, and, to some extent, intensity of sweet taste, accompanied by an intensification of specific vegetal notes from the added ingredient, particularly grass vegetation flavor, green leaf flavor, grassy taste, malt taste, and, in certain formulations, green walnut flavor/taste. The general trend is consistent with recent reports on functional chocolate showing that plant ingredients frequently confer “green,” “herbal,” “earthy,” or “grassy” notes, especially at medium and high addition levels [101].

The control formula and the low concentrations of barley powder addition led to high scores of cocoa butter flavor, powdered milk flavor, and sweet taste intensity, whereas at higher fortification levels, particularly 5 and 7%, vegetal and malty notes were emphasized. A recent study highlighted that the addition of plant-based powders to chocolate significantly altered color, rheology, and sensory perception [39,99].

Sweet taste intensity was among the descriptors with relatively high scores in all experimental treatments, though the slight decrease in the fortified formulations, which is technologically important because it suggests that the white chocolate matrix maintains the hedonic characteristics necessary for product acceptance upon the incorporation of plant ingredients. In this respect, the success of functional products depends on the formulation’s ability to preserve the dominant hedonic attributes, i.e., sweet, creamy, milky, after incorporating bioactive compounds; that is why chocolate is considered a suitable food matrix for functionalization, with the fat content and sweetness partially masking the less familiar notes of the fortifying ingredients [96].

Chocolate derived from cocoa butter developed some plant and malty notes more distinctly at high addition levels, while the milk butter derived product better preserved the milky and sweet character. The mentioned difference is presumably due to the influence of fat matrix and overall composition on the release of volatile compounds and taste perception. Recent studies reported that changing the lipid base altered the texture and malt but also the perceived intensity of dominant aromatic notes and the aftertaste of white chocolate [102,103].

In our research, the increase in the scores of “grassy,” “green leaf,” and “malt” directly reflects the sensory integration of green barley powder. Previous authors found that fortifying chocolate with high-value-added plant substances frequently resulted in a trade-off between improving nutritional value and deviating from the traditional sensory profile, thus highlighting the importance of optimizing the ingredient addition level [95,98].

In previous research [28], increases were recorded in aroma and flavor, thickness, color intensity, dryness and graininess in chocolate chip cookies with rising barley flour addition up to 70%. Moreover, the global texture and flavor acceptance was reduced by augmenting the integration of barley extract up to 10% [52]. In the latter respect, the time of melting chocolate during its mastication dynamically influences the perceived flavor intensity. Consequently, the longer time due to the presence of more solid particles because of the substitution of cocoa butter with barley fibrous material caused the decrease in sensory score [52].

Chocolate added with tea extract was slightly harder and had a more attractive color, compared to the untreated control [74].

At high concentrations of green barley powder integration, the sensory profile differed more remarkably from the classic model, thus arising the need for repositioning the product toward the functional chocolate segment, where consumers may tolerate or even appreciate unconventional notes associated with perceived nutritional benefits. Recent studies on cocoa-based functional foods and beverages demonstrated that the perceived health benefit can partially offset moderate sensory deviations, though the overall acceptability remains closely dependent on maintaining a pleasant consumption experience [104,105].

Overall, the sensory evaluation results confirm that green barley powder can be used to improve the health properties of white chocolate without negatively affecting consumer perception at moderate addition levels, which is important for the development of functional foods, as the commercial success of these products depends both on nutritional benefits and on sensory acceptability.

## 4. Conclusions

This research was aimed at producing white chocolate as a functional food by adding green barley powder to milk and cocoa butter at four concentrations (1%, 3%, 5% and 7%), compared to an untreated control.

Moderate additions of green barley powder, regardless of the type of fat phase used, enhanced fiber, proteins and mineral substances of white chocolate, thus justifying the functional food strategy, but high integrations compromised the visual profile and reduced its mechanical strength. From a legislative perspective, color and texture parameters do not represent numerical compliance criteria for the designation “white chocolate,” but they directly influence the product’s typical characteristics and its commercial acceptability.

Rising barley powder addition increased the antioxidant content and most of the vegetal flavor attributes, compared to the untreated white chocolate samples.

From the present research, it can be inferred that manufacturing innovative chocolate as a functional food obtained upon the integration of green barley powder, represents an interesting strategy to produce innovative aliment with highly health beneficial impact on the human organism. However, further studies with a larger panel are needed to confirm the findings related to sensory evaluation.

## Figures and Tables

**Table 1:** Chocolate recipes.

Type of Butter (TB) × Percentage of Green Barley Powder Addition (PA)	Powdered Milk (%)	Sugar (%)	Water (%)	Milk Butter or Cocoa Butter (%)	Vanilla Essence (%)	Green Barley Powder (%)
Chocolate from Milk Butter with no addition	35	30	16	18	1	-
CBMB1	34	30	16	18	1	1
CBMB3	32	30	16	18	1	3
CBMB5	30	30	16	18	1	5
CBMB7	28	30	16	18	1	7
Chocolate from Cocoa Butter with no addition	35	30	16	18	1	-
CBCB1	34	30	16	18	1	1
CBCB3	32	30	16	18	1	3
CBCB5	30	30	16	18	1	5
CBCB7	28	30	16	18	1	7

CBMB1: chocolate from 1% barley powder addition to milk butter; CBMB3: chocolate from 3% barley powder addition to milk butter; CBMB5: chocolate from 5% barley powder addition to milk butter; CBMB7: chocolate from 7% barley powder addition to milk butter; CBCB1: chocolate from 1% barley powder addition to cocoa butter; CBCB3: chocolate from 3% barley powder addition to cocoa butter; CBCB5: chocolate from 5% barley powder addition to cocoa butter; CBCB7: chocolate from 7% barley powder addition to cocoa butter.

**Table 2:** Chemical parameters of chocolate produced by adding green barley powder to milk butter and cocoa butter.

Type of Butter (TB) × Percentage of Green Barley Powder Addition (PA)	Dry Matter (%)	Soluble Solids (°Brix)	pH	Fat (%)	Proteins (%)	Fiber (%)	Ash (%)	Mineral Substances (%)
Chocolate from Milk Butter with no addition	88.3 a	20.5 a	6.66 ac	25.8 ab	13.9 d	0.30 g	2.45 b	6.95 c
CBMB1	88.2 a	20.3 a	6.64 bc	25.0 bd	14.3 cd	0.45 f	2.49 b	7.04 c
CBMB3	85.9 c	19.9 ab	6.58 de	23.6 df	14.7 ad	0.78 e	2.87 a	7.08 bc
CBMB5	85.4 c	19.4 ab	6.57 e	22.0 fg	15.4 ac	1.20 c	2.97 a	7.12 bc
CBMB7	84.6 d	19.0 b	6.55 de	21.0 g	15.6 ab	1.35 b	3.07 a	7.41 a
Chocolate from Cocoa Butter with no addition	88.5 a	20.5 a	6.70 a	26.7 a	14.5 bd	0.27 g	2.46 b	7.01 c
CBCB1	88.4 a	20.3 a	6.67 ab	26.3 ab	14.6 bd	0.49 f	2.53 b	7.07 c
CBCB3	87.6 b	20.0 ab	6.65 bc	25.3 ac	15.1 ac	0.72 e	2.60 b	7.22 ac
CBCB5	86.0 c	19.6 ab	6.62 cd	23.9 ce	15.5 ab	1.14 d	2.89 a	7.22 ac
CBCB7	84.8 d	19.2 b	6.57 de	22.5 eg	15.9 a	1.52 a	3.07 a	7.35 ab

CBMB1: chocolate from 1% barley powder addition to milk butter; CBMB3: chocolate from 3% barley powder addition to milk butter; CBMB5: chocolate from 5% barley powder addition to milk butter; CBMB7: chocolate from 7% barley powder addition to milk butter; CBCB1: chocolate from 1% barley powder addition to cocoa butter; CBCB3: chocolate from 3% barley powder addition to cocoa butter; CBCB5: chocolate from 5% barley powder addition to cocoa butter; CBCB7: chocolate from 7% barley powder addition to cocoa butter; within each column, mean values followed by different letters are significantly different at *p* ≤ 0.05 according to Duncan’s test.

**Table 3:** Color and textural characteristics of chocolate produced by adding green barley powder to milk butter and cocoa butter.

Type of Butter (TP) × Percentage of Green Barley Powder Addition (PA)	Color Components			F Max Compression (N)	F Max Cutting (N)
	L	a	b		
Chocolate from Milk Butter with no addition	83.7 b	−8.2 b	25.5 a	25.2 ab	355.1 a
CBMB1	65.7 c	−9.3 d	22.7 bc	24.6 ac	352.8 a
CBMB3	53.5 e	−9.4 d	21.0 d	22.5 cd	317.8 ac
CBMB5	50.2 f	−9.4 d	20.4 d	21.5 d	286.8 cd
CBMB7	43.7 gh	−9.5 d	17.6 e	19.0 e	278.5 d
Chocolate from Cocoa Butter with no addition	89.2 a	−7.0 a	26.7 a	25.7 a	334.1 ab
CBCB1	65.1 c	−8.4 bc	23.1 bc	25.6 a	332.2 ab
CBCB3	56.3 d	−8.9 cd	21.2 cd	25.4 ab	321.3 ac
CBCB5	45.7 g	−9.2 d	18.4 e	23.3 bd	308.8 bd
CBCB7	41.4 h	−9.3 d	15.9 f	22.4 d	292.9 cd

CBMB1: chocolate from 1% barley powder addition to milk butter; CBMB3: chocolate from 3% barley powder addition to milk butter; CBMB5: chocolate from 5% barley powder addition to milk butter; CBMB7: chocolate from 7% barley powder addition to milk butter; CBCB1: chocolate from 1% barley powder addition to cocoa butter; CBCB3: chocolate from 3% barley powder addition to cocoa butter; CBCB5: chocolate from 5% barley powder addition to cocoa butter; CBCB7: chocolate from 7% barley powder addition to cocoa butter; within each column, mean values followed by different letters are significantly different at *p* ≤ 0.05 according to Duncan’s test.

**Table 4:** Antioxidant activity and content of vitamin C and polyphenols in chocolate produced by adding green barley powder to milk butter and cocoa butter.

Type of Butter (TB) × Percentage of Green Barley Powder Addition (PA)	Antioxidant Activity (μmol Trolox eq. g^−1^)	Vitamin C (mg 100 g^−1^)	Polyphenols (mg 100 g^−1^)
Chocolate from Milk Butter with no addition	2.3 f	1.2 e	0.00 i
CBMB1	3.5 e	14.1 d	0.04 h
CBMB3	4.3 d	15.0 d	0.08 g
CBMB5	5.3 c	15.8 d	0.14 f
CBMB7	6.4 b	19.4 c	0.21 e
Chocolate from Cocoa Butter with no addition	3.4 e	1.9 e	0.21 e
CBCB1	4.3 d	15.0 d	0.24 d
CBCB3	5.5 c	20.7 c	0.29 c
CBCB5	6.8 b	30.9 b	0.37 b
CBCB7	8.0 a	42.8 a	0.43 a

CBMB1: chocolate from 1% barley powder addition to milk butter; CBMB3: chocolate from 3% barley powder addition to milk butter; CBMB5: chocolate from 5% barley powder addition to milk butter; CBMB7: chocolate from 7% barley powder addition to milk butter; CBCB1: chocolate from 1% barley powder addition to cocoa butter; CBCB3: chocolate from 3% barley powder addition to cocoa butter; CBCB5: chocolate from 5% barley powder addition to cocoa butter; CBCB7: chocolate from 7% barley powder addition to cocoa butter; within each column, mean values followed by different letters are significantly different at *p* ≤ 0.05 according to Duncan’s test.

**Table 5:** Content of carotenoids and chlorophylls in chocolate produced by adding green barley powder to milk butter and cocoa butter.

Type of Butter (TB) × Percentage of Green Barley Powder Addition (PA)	Carotenes (mg 100 g^−1^)	Lycopene (mg 100 g^−1^)	Xanthophylls (mg 100 g^−1^)	Chlorophyll a (mg 100 g^−1^)	Chlorophyll b (mg 100 g^−1^)
Chocolate from Milk Butter with no addition	1.33 g	2.58 e	1.61 h	1.7 de	1.3 e
CBMB1	1.52 g	2.94 d	2.25 g	2.5 d	2.1 d
CBMB3	2.64 e	3.40 c	2.36 fg	3.5 c	3.8 c
CBMB5	4.04 c	4.73 b	2.56 f	4.7 b	4.5 b
CBMB7	5.43 a	6.30 a	2.95 e	5.8 a	6.0 a
Chocolate from Cocoa Butter with no addition	1.32 g	1.92 g	2.15 g	1.7 de	1.5 e
CBCB1	1.65 g	2.27 f	3.39 d	2.6 d	2.5 d
CBCB3	2.19 f	2.92 d	3.68 c	3.4 c	3.8 c
CBCB5	3.39 d	3.57 c	3.94 b	4.6 b	4.8 b
CBCB7	4.55 b	5.00 b	4.41 a	5.6 a	6.0 a

CBMB1: chocolate from 1% barley powder addition to milk butter; CBMB3: chocolate from 3% barley powder addition to milk butter; CBMB5: chocolate from 5% barley powder addition to milk butter; CBMB7: chocolate from 7% barley powder addition to milk butter; CBCB1: Chocolate from 1% barley powder addition to cocoa butter; CBCB3: chocolate from 3% barley powder addition to cocoa butter; CBCB5: chocolate from 5% barley powder addition to cocoa butter; CBCB7: chocolate from 7% barley powder addition to cocoa butter; within each column, mean values followed by different letters are significantly different at *p* ≤ 0.05 according to Duncan’s test.

**Table 6:** Mineral composition of chocolate produced by adding green barley powder to milk butter and cocoa butter.

Type of Butter (TB) × Percentage of Green Barley Powder Addition (PA)	K	Ca	Mg	Na	P	Cu	Zn	Fe
			mg 100 g^−1^					
Chocolate from Milk Butter with no addition	161.1	252.0	88.4 f	162.6 c	9.1 de	0.09 e	1.14 f	0.00 h
CBMB1	160.8	252.8	90.8 ef	167.8 bc	9.1 de	0.09 e	1.20 ef	0.34 g
CBMB3	156.5	253.9	93.7 df	173.6 bc	9.7 cd	0.10 d	1.38 d	2.63 e
CBMB5	152.3	255.6	100.1 cd	180.2 ab	10.4 bc	0.11 c	1.59 c	3.50 c
CBMB7	148.3	257.2	116.3 a	188.6 a	10.8 b	0.12 b	1.90 a	4.06 b
Chocolate from Cocoa Butter with no addition	160.8	244.5	91.8 df	167.1 bc	7.9 f	0.09 e	1.18 f	0.00 h
CBCB1	159.6	247.0	94.3 df	170.9 bc	8.4 ef	0.09 e	1.28 e	0.50 g
CBCB3	158.8	251.9	99.1 ce	173.3 bc	9.0 de	0.10 cd	1.39 d	2.22 f
CBCB5	157.8	256.0	104.9 bc	176.5 ac	10.4 b	0.12 b	1.52 c	3.17 d
CBCB7	156.2	259.4	109.4 ab	179.7 ab	12.4 a	0.15 a	1.72 b	4.36 a
	n.s.	n.s.						

CBMB1: chocolate from 1% barley powder addition to milk butter; CBMB3: chocolate from 3% barley powder addition to milk butter; CBMB5: chocolate from 5% barley powder addition to milk butter; CBMB7: chocolate from 7% barley powder addition to milk butter; CBCB1: chocolate from 1% barley powder addition to cocoa butter; CBCB3: chocolate from 3% barley powder addition to cocoa butter; CBCB5: chocolate from 5% barley powder addition to cocoa butter; CBCB7: chocolate from 7% barley powder addition to cocoa butter; n.s.: not significant; within each column, mean values followed by different letters are significantly different at *p* ≤ 0.05 according to Duncan’s test.

**Table 7:** Statistically significant differences between the experimental treatments in terms of the sensory features of chocolate added with green barley powder.

TP × PA	EA	AS	EC	IC	F	T	GP	AP	BP	TP	MP	BP	SP	HP	CBF	AFF	PMF	GVF	GLF	GWF	PMT	CBT	AFT	GT	BT	IST	GWT	MT
CMBNA	b	b	b	b	c	a	a	b	b	b	ab	a	b	a	d	a	a	e	e	f	a	c	a	f	f	a	d	b
CBMB1	b	b	b	b	c	a	ac	b	b	b	ab	a	b	a	d	b	ab	c	cd	de	ab	c	ab	cd	ef	ab	cd	ab
CBMB3	ab	ab	ab	b	c	a	ac	b	b	b	ab	ac	ab	a	d	b	ab	b	bc	bd	ab	c	bc	cd	ef	bc	bc	ab
CBMB5	ab	ab	ab	ab	c	ab	bc	a	b	a	b	ac	a	ab	d	bc	bc	b	ab	ac	bc	c	bc	ab	de	bc	ab	a
CBMB7	ab	a	a	a	bc	b	c	a	a	a	b	bc	a	c	d	bc	bc	a	a	a	bc	c	c	a	cd	c	a	a
CCBNA	b	b	b	b	a	a	a	b	b	b	a	ab	ab	a	c	cd	f	d	d	ef	bc	a	d	e	a	ab	cd	b
CBCB1	b	b	b	ab	a	ab	ab	b	b	b	a	ac	ab	a	c	de	ef	cd	cd	de	bc	a	d	e	ab	ab	cd	ab
CBCB3	ab	ab	ab	ab	ab	ab	ac	b	b	b	a	ac	ab	ab	bc	ef	de	c	bc	ce	cd	ab	d	de	bc	ac	bc	ab
CBCB5	ab	ab	ab	ab	ac	ab	ac	a	b	a	ab	bc	ab	bc	ab	f	cd	b	ab	bd	cd	ab	d	bc	cd	bc	bc	a
CBCB7	a	a	a	a	bc	b	bc	a	a	a	b	c	ab	c	a	g	bc	a	a	ab	d	c	d	bc	de	c	ab	a

CMBNA: Chocolate from milk butter with no addition; CBMB1: Chocolate from 1% barley powder addition to milk butter; CBMB3: Chocolate from 3% barley powder addition to milk butter; CBMB5: Chocolate from 5% barley powder addition to milk butter; CBMB7: Chocolate from 7% barley powder addition to milk butter; CCBNA: Chocolate from cocoa butter with no addition; CBCB1: Chocolate from 1% barley powder addition to cocoa butter; CBCB3: Chocolate from 3% barley powder addition to cocoa butter; CBCB5: Chocolate from 5% barley powder addition to cocoa butter; CBCB7: Chocolate from 7% barley powder addition to cocoa butter; EA: exterior appearance; AS: appearance in section; EC: exterior color; IC: inner color; F: flavor; T: texture; GP: general perception; AP: acid perception; BP: bitter perception; TP: tasteless perception; MP: mastication perception; BP: bitter perception; SP: stickiness perception; HP: hardness perception; CBF: cocoa butter flavor; AFF: animal fat flavor; PMF: powdered milk flavor; GVF: grass vegetation flavor; GLF: green leaf flavor; GWF: green walnut flavor; PMT: powdered milk taste; CBT: cocoa butter taste; AFT: animal fat taste; GT: grassy taste; BP: bitter taste; IST: intensity of sweet taste; GWT: green walnut taste; MT: malt taste. Within each column, different letters are associated with significant differences according to Duncan’s test at *p* ≤ 0.05.

## Data Availability

The original contributions presented in this study are included in the article. Further inquiries can be directed to the corresponding authors.
